# A fragmented alignment method detects a putative phosphorylation site and a putative BRC repeat in the *Drosophila melanogaster* BRCA2 protein

**DOI:** 10.12688/f1000research.2-143.v2

**Published:** 2013-10-07

**Authors:** Sandeep Chakraborty

**Affiliations:** 1 Department of Biological Sciences, Tata Institute of Fundamental Research, Mumbai, 400 005, India

## Abstract

Mutations in the BRCA2 tumor suppressor protein leave individuals susceptible to breast, ovarian and other cancers. The BRCA2 protein is a critical component of the DNA repair pathways in eukaryotes, and also plays an integral role in fostering genomic variability through meiotic recombination. Although present in many eukaryotes, as a whole the *BRCA2* gene is weakly conserved. Conserved fragments of 30 amino acids (BRC repeats), which mediate interactions with the recombinase RAD51, helped detect orthologs of this protein in other organisms. The carboxy-terminal of the human BRCA2 has been shown to be phosphorylated by checkpoint kinases (Chk1/Chk2) at T3387, which regulate the sequestration of RAD51 on DNA damage. However, apart from three BRC repeats, the *Drosophila melanogaster* gene has not been annotated and associated with other functionally relevant sequence fragments in human BRCA2. In the current work, the carboxy-terminal phosphorylation threonine site (E=9.1e-4) and a new BRC repeat (E=17e-4) in *D. melanogaster *has been identified, using a fragmented alignment methodology (FRAGAL). In a similar study, FRAGAL has also identified a novel half-a- tetratricopeptide (HAT) motif (E=11e-4), a helical repeat motif implicated in various aspects of RNA metabolism, in Utp6 from yeast. The characteristic three aromatic residues with conserved spacing are observed in this new HAT repeat, further strengthening my claim. The reference and target sequences are sliced into overlapping fragments of equal parameterized lengths. All pairs of fragments in the reference and target proteins are aligned, and the gap penalties are adjusted to discourage gaps in the middle of the alignment. The results of the best matches are sorted based on differing criteria to aid the detection of known and putative sequences. The source code for FRAGAL results on these sequences is available at https://github.com/sanchak/FragalCode, while the database can be accessed at www.sanchak.com/fragal.htm l.

## Introduction

The breast cancer susceptibility protein BRCA2, first identified in 1995 ^1^, is a critical recombinase regulator ^2^ that ensures genomic stability through high fidelity repair ^3, 4^ of double stranded breaks (DSB) and prevents stalled replication forks from replicating ^5^ in the DNA. The primary recombinase in BRCA2 repair of DSB through homologous recombination is the RAD51 protein, belonging to the conserved RecA/RAD51 family ^6^, that binds to the BRCA2 protein at various segments of ~30 amino acids (BRC repeats) ^7, 8^, and in the C-terminal region in most vertebrates ^9, 10^. Checkpoint kinases phosphorylate a serine ^9^ and a threonine ^10^ at the carboxy-terminal region of BRCA2, thereby regulating its interaction with RAD51. BRCA2 also plays a key role in fostering genomic variability through meiotic recombination ^11, 12^, although a different recombinase (DMC1) is implicated in this pathway in mammalian species ^13^.

The BRC repeats have helped identify BRCA2 orthologs in various eukaryotic species ^14^. Functional characterization of this gene in *Drosophila melanogaster* has demonstrated its interaction with RAD51, and a critical role in mitotic and meiotic DNA repair as well as homologous recombination ^11, 15^. The copy number of the BRC repeats differs considerably. The BRCA2 homolog in *Ustilago maydis* (a yeast like fungus) has a single BRC repeat ^16^, the *D. melanogaster* homolog contains only three (known) repeats ^14^, while there are eight repeats in the human BRCA2 gene ^7^. Even among the *Drosophila* genus, the range of BRC repeat numbers is varied - the *D. melanogaster* species has only three repeats, while *D. persimilis* and *D. pseudoobscura* have up to eleven repeats ^17^. RAD51 shows varying affinity for the different BRC motifs ^18, 19^. This difference in repeat numbers in *Drosophila* has raised doubts whether ‘this higher repeat number is real or a genome mis-assembly artifact’ ^20^, and also led to speculation on the evolution of these closely related organisms ^17, 20^. Any such hypothesis would need to be revisited if a new BRC motif were to be identified in *D. melanogaster*.

In the current work, the putative threonine phosphorylation site for checkpoint kinases (Chk1/Chk2) (E=9.1e-4) and a new BRC repeat (E=17e-4) in *D. melanogaster* has been identified, using a fragmented technique for the pairwise alignment of two sequences (FRAGAL). The reference and target sequences are sliced into fragments of equal parameterized length X, sliding along the sequence in intervals of length Y, such that Y is less than X. Thus, the slices have overlaps. An alignment of all pairs of slices in the reference and target proteins is done using the global alignment program ‘needle’ ^21^ from the EMBOSS suite ^22^. The gap penalties are adjusted to discourage gaps in the middle of the alignment. The results of the best matches are sorted based on differing criteria to aid the detection of known and putative sequences. In order to establish the generic nature of the FRAGAL methodology, the detection of a new half-a-tetratricopeptide (HAT) repeat sequence (E=11e-4) in a nucleolar RNA-associated protein (Utp6) from *Saccharomyces cerevisiae* is also reported. HAT is a helical repeat motif implicated in various aspects of RNA metabolism ^23, 24^. The characteristic three aromatic residues with a conserved spacing are observed in this new HAT repeat, further strengthening my claim ^25^.

Existing methods for detecting functional motifs in a given protein sequence have been unable to detect these putative sites. For example, meta servers ( http://myhits.isb-sib.ch/cgi-bin/motif_scan, http://www.ebi.ac.uk/Tools/pfa/iprscan/, http://www.genome.jp/tools/motif/) for detecting motifs in a protein have been unable to detect the sites identified using the FRAGAL methodology. These meta servers use one or more motif databases ^26– 30^. Not all known BRC repeats have a low E-value when aligned with the new BRC repeat. For example, the first BRC repeat in hBRCA2 when aligned to the new dmBRCA2 repeat has an E=0.04, much more than the E=17e-4 observed for the fourth repeat, which is the one I report here. Ideally, if one took all the BRC repeats and did a search in the dmBRCA2 sequence, this new repeat would be reported. Essentially, this is what FRAGAL does - albeit implicitly, by automatically fragmenting the sequence. The same logic applies to the HAT repeat, where the sequences are more varied and thus the choice of the repeat would effect the detection of new motifs.

Spliced alignment techniques have frequently been adopted in the precise identification of eukaryotic gene structures, and in gene assembly. These methods try to solve the exon assembly problem by searching the exon sequence space to find the best fit to known proteins ^31, 32^. While these methods use graph algorithms to solve the computationally difficult problem of exon chaining, FRAGAL does the converse of finding best matches in known exon chains (i.e. protein sequences).

It is fair to mention that the FRAGAL method is much more computationally intensive than the above mentioned methods. At the same time, FRAGAL makes no assumption of any knowledge of the conserved regions (either the sequence or their position). The choice of the fragment length in FRAGAL depends on the length of repeats that is expected to be present in the protein. Since both repeats (BRC and HAT) discussed in this manuscript are around ~30 amino acid long, I have chosen a fragment length of 50. A larger fragment length might mask the similarity in the core region due to variations in the non-critical regions, whereas a smaller fragment would match irrelevant portions and thus increase false positives.

The significant conservation of the DNA repair and checkpoint pathways in flies and higher organisms ^33^, the advanced genetic tools available for *Drosophila*, and the viability of the *Drosophila* BRCA2 null mutants in contrast to mammalian mutants ^34^ establishes *Drosophila* as a model organism for studying these pathways ^35^. Significant divergence of key conserved sequences proves to be a serious hurdle for alignment techniques to annotate and associate the conserved sequences in the human BRCA2 to the *Drosophila* BRCA2 ^36^. Thus, a generic methodology, applicable to distantly evolutionary related proteins like BRCA2 and nucleolar RNA-associated proteins is presented. The methodology has been validated by the identification of two novel functionally relevant sites in the BRCA2 protein from *D. melanogaster*, and a HAT repeat in Utp6 from *S. cerevisiae*.

## Materials and methods

**Figure S1.**
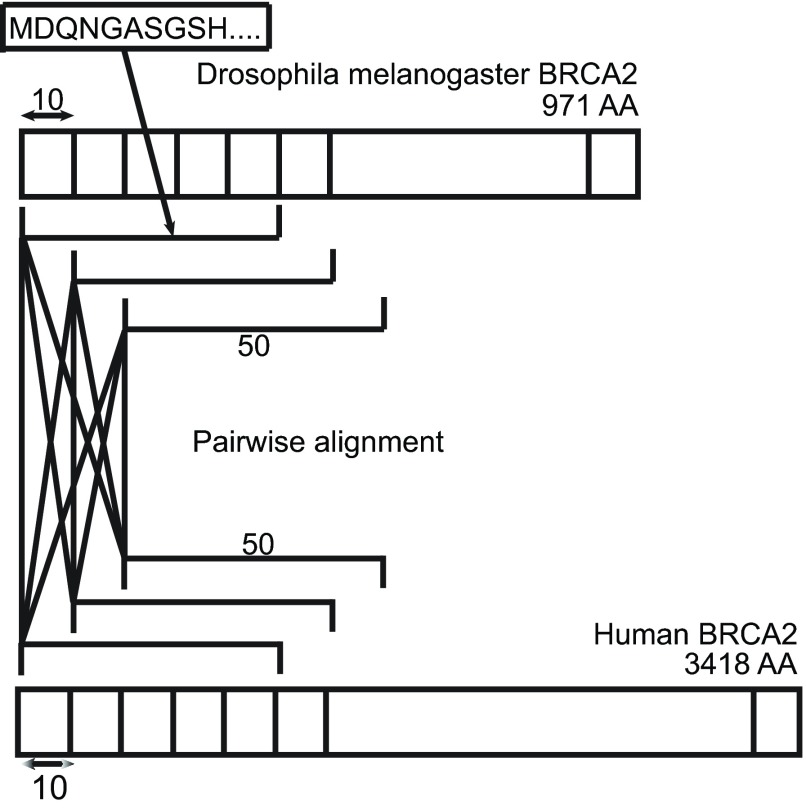
FRAGAL methodology. The BRCA2 protein sequences from *Drosophila melanogaster* and humans are split into fragments of parameterized length (50 in this case), at a parameterized interval (10 in this case). All pairs of fragments are aligned, and the results stitched such that there are no overlap in any given match and the order of the match is not interspersed. The alignment is done using the global alignment program ‘needle’ from the EMBOSS suite, and the gap penalties are set to 25 to discourage gaps in the middle of the alignment.

The FRAGAL methodology is shown in Supplementary Figure 1.

**Algorithm 1: FRAGAL() - A fragmented alignment method**

**Input**: *A*: Query sequence

**Input**: *B*: Target sequence

**Input**: *X*: Length of fragments

**Input**: *Y*: Length of sliding window

**Input**: *gapopen*: Length of fragments

**Input**: *gapextend*: Length of fragments

**Output**: *ϕ _fragments_*: Matching fragments sorted in terms of higher FRscores

**begin**

  
*ϕ* *_Afrag_* = FragementSequenceIntoOverlappingSegments( *A, X, Y*);

  
*ϕ* *_Bfrag_* = FragementSequenceIntoOverlappingSegments( *B, X, Y*);

  // Create priority queue, based on FRscore

  
*ϕ* *_fragments_* = ∅ ;

  
**for** *each fragemnt A _i_ in* *ϕ* *_Afrag_* **do**

     
**for** *each fragemnt B _j_ in* *ϕ* *_Bfrag_* **do**

        // See methods section for FRscore

        
*FRscore _ij_* = RunNeedle( *A _i_,B _j_, gapopen, gapextend*);

        Insert( *ϕ _fragments_,* *FRscore _ij_*);

     
**end**

  
**end**

  
**return** ( *ϕ _fragments_*);

**end**

The sequences are split into fragments of X amino acids, with the starting indices sliding across the sequence length in steps of Y amino acids (SI.A.fasta and SI.B.fasta in Data Files). The score for each match is computed as shown in Equation 1. FRscore is intended to give more weightage to identical residues in the alignment.

*FRscore* = 1/3 * *%onlySimilarity* + 2/3 * *%identity*;         (1)

One sorting criteria is to rank the matches based on the best average score, while another takes the cumulative score of a stretch of fragment matches. Stretches of fragments are stitched while ensuring the slices in the sequences are in an increasing order and non-overlapping. The best average criteria will typically select single fragments, while the cumulative scoring criteria will bring forth longer conserved regions.

The threshold for sequence similarity for each fragment is parameterized, and set to 30% in the default mode. A large threshold will exclude more relevant matches, while a smaller threshold might include more false positives. The pairwise alignment for each fragment pair is done by a global alignment program ‘needle’ from the EMBOSS suite ^21, 22^. The parameters are set as follows - matrix=BLOSUM62, Gap penalty=25.0 and Extend penalty=0.5. The gap penalty is increased from the default value of 10 to ensure that gaps are discouraged in the middle of the alignment. Single deletions or insertions are rarely expected in conserved fragments. However, once ‘needle’ has aligned the sequences based on the this penalty, gaps should not have a penalty. It is for this very reason that I have introduced the FRscore as a metric to measure quality of alignment, which creates a weighted score of the %identity and %similarity ( Equation 1).

**Figure S2.**
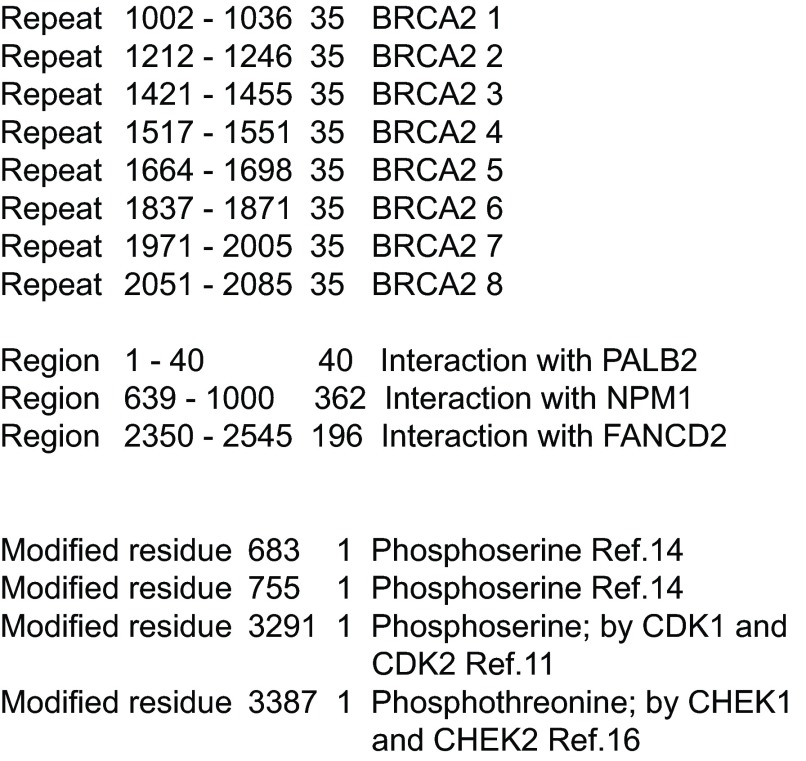
Annotating the hbrca sequence. The syntax is similar to the one used in the UNIPROT accession site.

The user is allowed to specify an annotation file for a given protein sequence using the uniprot accession syntax ( Supplementary Figure 2). The results from FRAGAL can be filtered based on this annotation, and this provides a easier way to manually inspect and annotate corresponding segments in a query protein sequence.

The FRAGAL package is written in Perl on Ubuntu. Hardware requirements are modest - all results here are from a simple workstation (2GB RAM). The source code for FRAGAL results on these sequences is available at https://github.com/sanchak/FragalCode, while the database can be accessed at www.sanchak.com/fragal.html. The multiple sequence alignment was done using ClustalW ^37^. PHYML has been used to generate phylogenetic trees from these alignments, which is based on the method of maximum likelihood ^38^. The method searches for a tree with the highest probability or likelihood that, given a proposed model of evolution and the hypothesized history, would give rise to the observed data set. The alignment and cladograms images were generated using Seaview ^39^. E-values and z-scores have been computed using the Protein Information Resource ( http://pir.georgetown.edu/pirwww/search/pairwise.shtml) ^40^.

UPDATE 1: BRCA2 sequence fragments and database of the output of FRAGAL for BRCA2 and HAT repeats for different organisms SI.A.fasta: Fragments of the Drosophila melanogaster BRCA2 sequence of size 50 amino acids. SI.B.fasta : Fragments of the human BRCA2 sequence of size 50 acids.
UPDATED DATABASE. Updated browsable database of the output of FRAGAL for BRCA2, HAT and two new repeats - BIR and TPR for different organisms. The results have been generated by varying two parameters - length of the fragments and the threshold %similarity value for a significant match in a fragment pair. The top level html file is fragal.html.

## Results

### Breast cancer susceptibility protein BRCA2

The *D. melanogaster* gene (CG30169) ^41^ encodes a 971 amino acid protein (dmBRCA2, Uniprot Accession:Q9W157), and contains three BRC repeat units (conserved sequences of ~30 amino acids that binds to RAD51) ^8, 14^. In contrast, the human BRCA2 gene product (hBRCA2, Uniprot Accession:P51587) is 3418 amino acids long and contains eight BRC repeats ^7^. Further, the hBRCA2 protein is annotated for several sites phosphorylated by checkpoint kinases, which regulate its interaction with RAD51 ^9, 10^. FRAGAL was run on the dmBRCA2 and hBRCA2 sequences. Table 1 shows the best matches obtained using two different sorting criteria - best average FRscore (see Methods) and best average %similarity - either when the match in hbrca2 is known to be conserved ( Table 1A) based on an user defined input file ( Supplementary Figure 2) or otherwise ( Table 1B).

**Table 1.** FRAGAL results for aligning the BRCA2 protein sequences from *Drosophila melanogaster* (A) (Accession:Q9W157) and humans (B) (Accession:P51587): The results are filtered out if the fragment in the hBRCA2 sequence is not marked as conserved ( Supplementary Figure 2). This filtering helps in removing already annotated sequences, thus making it easier to observe new sequences. Thus, there are some missing ranks. Multiply index with 10 to get sequence starting position in original sequence. A91 refers to the sequence starting at 910 in A and going till 959, since the fragmenting length is 50. The A91-B337 match corresponds to the phosphorylation site of checkpoint kinases in the carboxy-terminal of BRCA2, while the match A61-B151 (not shown in Table, FRscore=53), corresponds to the new BRC repeat identified in *D. melanogaster*.

Rank	FRscore	Matches
1	73.5	A91-B337
3	64.7	A87-B197
4	64.2	A67-B152
5	64	A87-B194
6	64	A65-B140
9	61.7	A57-B167,A87-B197
10	61.4	A67-B100
11	61.4	A69-B199,A74-B204
12	61.3	A89-B335
14	60.1	A75-B152

**Figure 1.**
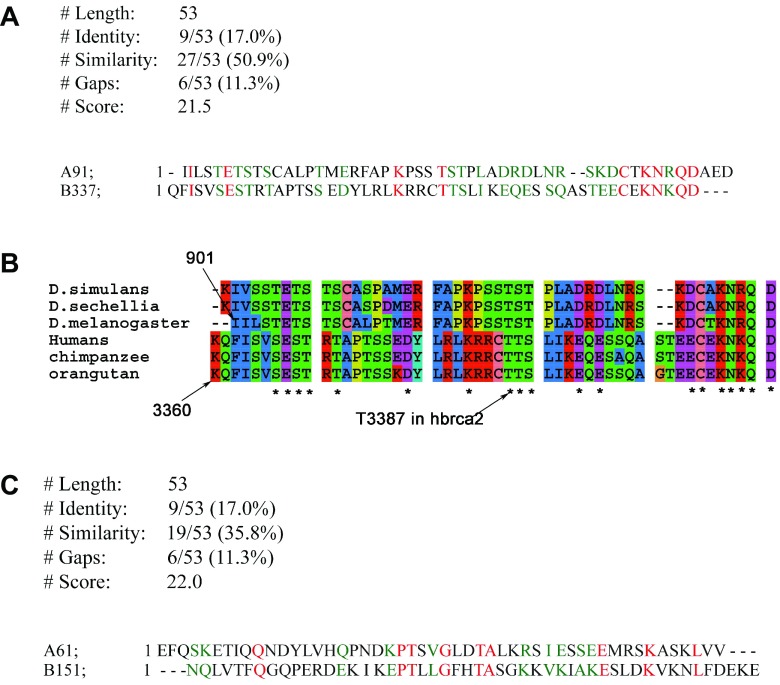
Fragment alignment using ‘needle’ from the EMBOSS suite ^21, 22^ of previously unknown conserved, and functionally relevant, sequences in dmBRCA2. (red for identity, green for similarity). ( **a**) Putative phosphorylation site by checkpoint kinases in the carboxy-terminal of hBRCA2. The threonine that is phosphorylated is highlighted (T3387 in hBRCA2 and T926 in dmBRCA2) (E=0.00091, Zscore=100). ( **b**) Conserved sequence in the carboxy-terminal of the BRCA2 protein sequence: Checkpoint kinases Chk1 and Chk2 phosphorylate threonine 3387 in hbrca2, and is seen to be conserved in the mammalian and *Drosophila* species (T926 in *dmBRCA2).* ( **c**) Putative BRC repeat identified by the similarity of fragment 61 (634–664:LDTALKRSIESSEEMRSKASKLVVVDTTMR) in *D. melanogaster* to the BRC4 repeat in hBRCA2 (1517–1551) (E=0.0017, Zscore=95) (red for identity, green for similarity).

***Detecting the threonine phosphorylation site in the carboxy-terminal region of dmBRCA2.*** Table 1 shows a significant match (E=9.1e-4, Z-score=100) between fragment 91 of dmBRCA2 to the fragment 337 in hBRCA2, which contains the T3387 that is phosphorylated by the checkpoint kinases Chk1 and Chk2. Z-scores above a value of 8 are considered to be significant ^42^. The alignment shows that the T3387 corresponds to the T926 of dmBRCA2 ( Figure 1a). The conservation of this region in the *Drosophila* and mammalian species is demonstrated by the multiple sequence alignment of three organisms from each species ( Figure 1b). The highly conserved columns in the alignment are highlighted using an asterisk, and can be used to define a Prosite motif ([ST]-E-[ST][ST]-x-[ST]-x(6)-[ED]-x(4)-K-x(4)-[ST]-[ST]-[ST]-x(3)-[DE]-[DE]) ^27^. Either this motif or FRAGAL alignments failed to detect this site in other species distant from *Drosophila* or mammals ( *Ustilago maydis* and *Caenorhabditis elegans*).

**Figure 2.**
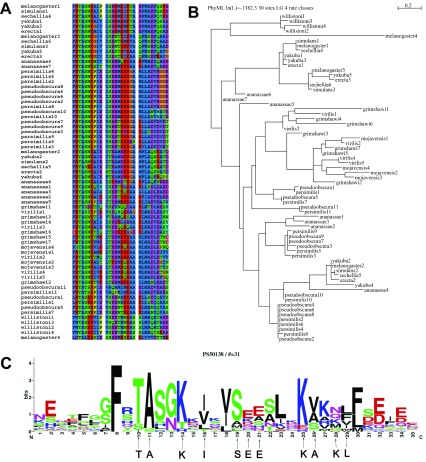
New BRC repeat identified by FRAGAL in *Drosophila melanogaster*. ( **a**) The multiple alignment for this new sequence (634–664:LDTALKRSIESSEEMRSKASKLVVVDTTMR) (using ClustalW) highlighted as melanogaster4 and other sequences compared previously in ^17^. This putative sequence is more closely related to the sequences in *D. willistoni* than other members of the genus. ( **b**) The phylogenetic tree (using PHYML) gives a graphical representation of the relation of the various repeats in the *Drosophila* genus, corroborating the closer relation of the new BRC repeat to *D. willistoni.* ( **c**) Alignment of the new BRC repeat to the sequence logo of the Prosite BRCA2 repeat profile PS50138.

**Figure S3.**
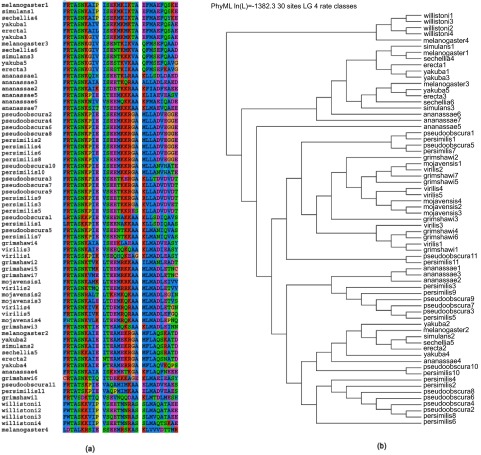
New BRC repeat identifed by FRAGAL in *Drosophila melanogaster* aligned using MAFFT. ( **a**) The multiple alignment for this new sequence (634-664:LDTALKRSIESSEEMRSKASKLVVVDTTMR) (using ClustalW) marked as melanogaster4 and other sequences compared previously in ^17^, using MAFFT for doing multiple sequence alignment. This putative sequence is more closely related to the sequences in *D. willistoni* than other members of the genus, as shown by the alignment done using ClustalW. ( **b**) The phylogenetic tree corroborates the closer relation of the new BRC repeat to *D. willistoni*, similar to the ClustalW results.

***Detecting an additional BRC repeat in Drosophila melanogaster.*** The correct identification of the three BRC repeats in *D. melanogaster* is seen by the significant scores of the FRscore matches of A67-B152 (64), A57-B100 (60) and A75-B152 (60) ( Table 1). A significant alignment (E=17e-4, Z-score=95) between A61-B151 (35.8%similarity and 17%identity) ( Figure 1c) was also observed. This sequence (634–664:LDTALKRSIESSEEMRSKASKLVVVDTTMR) is now added to the list of sequences previously studied in the *Drosophila* genus ^17^. The multiple sequence alignment (obtained using ClustalW ^37^) ( Figure 2a) and phylogenetic trees (obtained using PHYML ^38^) ( Figure 2b) shows that this new BRC repeat is more related to *D. willistoni* than other organisms in the *Drosophila* genus. A detailed molecular phylogeny of Drosophilid species has noted that the subgenus *Sophophora* is ‘divided into *D. willistoni* and the clade of *D. obscura* and *D. melanogaster* groups’, possibly indicating the source of this BRC repeat that has been conserved between *D. willistoni* and *D. melanogaster* ^43^. The same inference is drawn when we use a different multiple alignment tool like MAFFT ( http://mafft.cbrc.jp/alignment/software/) ^44^ ( Supplementary Figure 3). An iterative methodology, similar to PSI-BLAST (Position-Specific Iterative Basic Local Alignment Search Tool) ^45^, can be automated to generate comprehensive motifs spanning distant species. The conservation of many key residues in this sequence fragment, as shown by comparing it to the sequence logo of the Prosite BRCA2 profile (PS50138) ( Figure 2c) strongly suggests that this is a putative BRC repeat. However, it must be emphasized that such repeats are to be considered putative until verified experimentally ^46, 47^.

### Half-a-tetratricopeptide (HAT) motif

**Figure 3.**
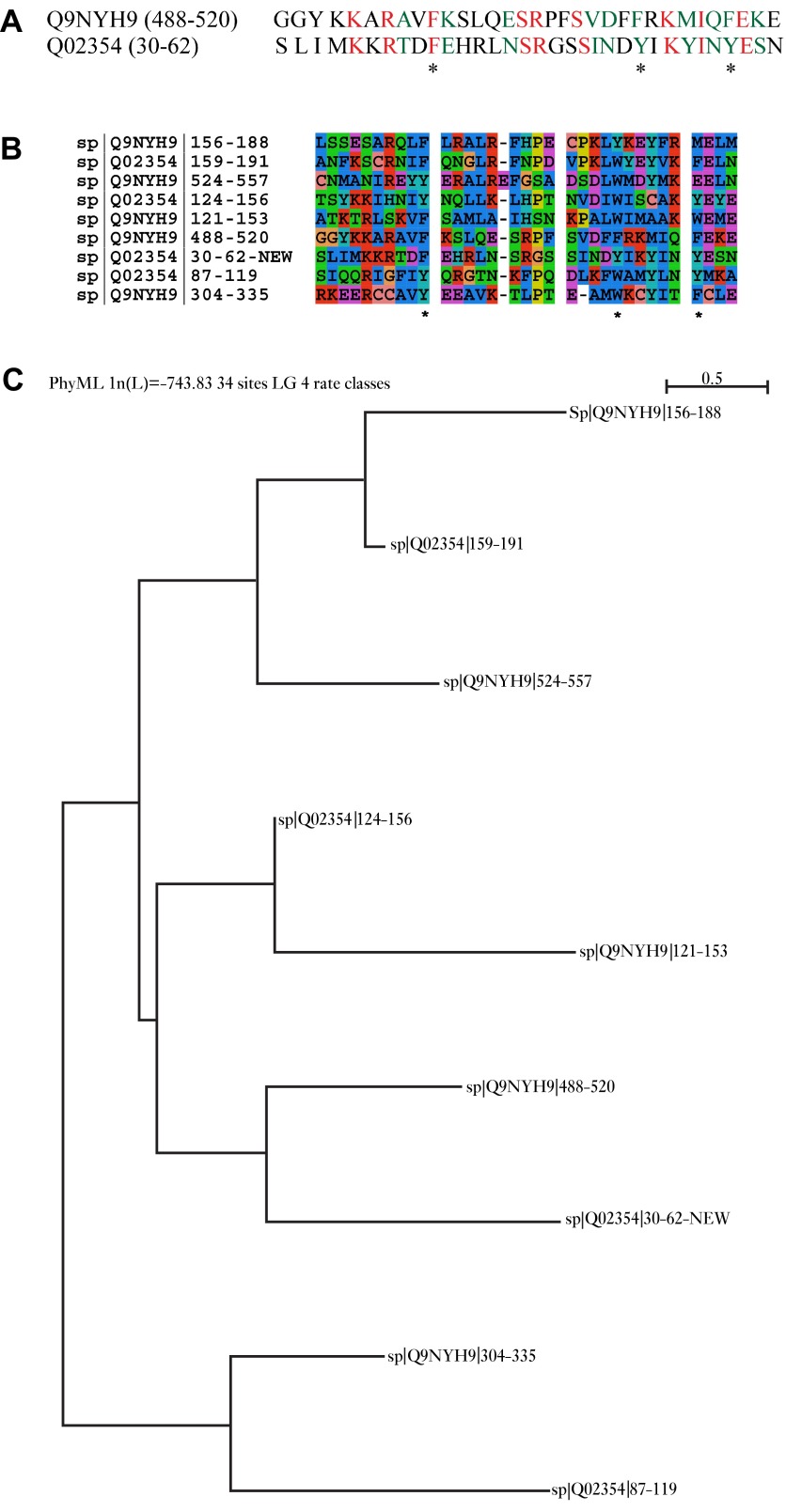
New Half-a-tetratricopeptide (HAT) motif identified by FRAGAL in *Saccharomyces cerevisiae*. ( **a**) Pairwise alignment of a previously unannotated HAT motif in *S. cerevisiae* (E=11e-4, Z-score=116) (red for identity, green for similarity). ( **b**) The multiple alignment for this new sequence (using ClustalW) with other HAT motifs in *S. cerevisiae* and humans shows large variation amongst HAT sequences even within the same organism. The conserved spacing of the aromatic residues are also highlighted. ( **c**) The phylogenetic tree (using PHYML) shows that certain HAT repeats are more similar to HAT repeats from other organisms than to other HAT repeats in its own sequence.

**Figure S4.**
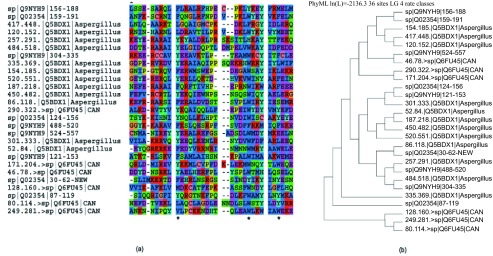
Including more proteins having the HAT motif. ( **a**) The multiple alignment for this new sequence (using ClustalW) with other HAT motifs in *S. cerevisiae*, humans, *Aspergillus nidulans* and *Candida glabrata* shows large variation amongst HAT sequences even within the same organism. The conserved spacing of the aromatic residues remains the same. ( **b**) Similar to the case when fewer organisms were included, the phylogenetic tree (using PHYML) shows that certain HAT repeats are more similar to HAT repeats from other organisms than to other HAT repeats in its own sequence.

HAT is a helical repeat motif implicated in various aspects of RNA metabolism and in protein-protein interactions ^23, 24^. These repeats are characterized by three aromatic residues with a conserved spacing ^25^. A variable number of HAT repeats (9 to 12) are found in different proteins. Figure 3a shows a novel HAT repeat (E=11e-4, Z-score=116) detected in a nucleolar RNA-associated protein (Utp6) from *Saccharomyces cerevisiae* (Uniprot Accession:Q02354) by comparing it to HAT repeats from a human nucleolar RNA-associated protein (Uniprot Accession:Q9NYH9). Q9NYH9 has five annotated HAT repeats (121–153, 156–188, 304–335, 488–520 and 524–557), while Q02354 has three HAT repeats (87–119, 124–156 and 159–191). The new HAT sequence identified in Q02354 (SLIMKKRTDFEHRLNSRGSSINDYIKYINYESN) is from position 30 to 62. It can be seen from the multiple sequence alignment that this sequence has the desired aromatic residues at the proper spacing, a requisite for being considered a HAT repeat ( Figure 3a and Figure 3b). Further, the MSA shows large variation amongst HAT sequences even within the same organism ( Figure 3b). Finally, Figure 3b and Figure 3c shows that certain HAT repeats are more similar to HAT repeats from other organisms than to other HAT repeats in its own sequence. Supplementary Figure 4 shows the alignment and phylogenetic tree when we include more proteins having HAT repeats from organisms closely related to *S. cerevisiae* like *Aspergillus nidulans* and *Candida glabrata*, corroborating the large variation among repeats even within the same organism and that often HAT repeats across organisms show more similarity.

### Database for aligning different pairs of BRCA2

A database ( www.sanchak.com/fragal.html) which lists the results for the fragmented alignment of various proteins with BRC and HAT repeats sequences has been created. The results have been generated by varying two parameters - length of the fragments and the threshold %similarity value for a significant match in a fragment pair. As mentioned above, the results are presented in two formats - best cumulative score and best average score.

## Discussion

Genetic evolution over large time spans often leaves little trace of kinship in different organisms, even when the functional roles of the genes remains conserved. A relevant example is the *BRCA2* gene which, although present in many eukaryotes, is weakly conserved ^48^. The BRCA2 protein plays a major role in maintaining genomic stability, fostering genetic variability and also has other cellular functions ^2, 49^. Individuals with germline mutations in the *BRCA2* gene are at significantly greater risk to a wide range of cancers ^50, 51^. This is supposed to be primarily due to the instability in chromosome structure and number induced by functional aberrations in BRCA2 ^52^. Conserved fragments of ~30 amino acids (BRC repeats) ^7^ that mediates the interaction of BRCA2 with the RAD51 recombinase ^53^ have been instrumental in identifying BRCA2 orthologs in other species ^14, 16^. The BRCA2 protein in the *Drosophila* genus assumes significance in this context owing to the advanced tools available for *Drosophila* genetics ^35^, and has been functionally characterized recently ^11, 15^.

However, weak sequence conservation in this gene has proven to be an impediment in associating experimentally proven functionally relevant gene fragments in humans and *Drosophila*. The variability in the number of BRC repeats even within the *Drosophila* species has provided fodder for further speculation on the evolution of this gene ^17, 20^. The detection of a new BRC repeat would necessitate the reevaluation of such hypotheses.

Apart from the BRC repeats, RAD51 interacts with BRCA2 in the carboxy-terminal, and this interaction is modulated by checkpoint kinases ^9, 10^. Since the introduction of BRC repeats in the cell inhibits the formation of RAD51 nucleoprotein filaments ^8^, a model has been suggested whereby RAD51 binds to both the BRC repeats and the carboxy-terminal in undamaged cells, and DNA damage triggers the release of the carboxy-terminal bound RAD51 via the phosphorylation of a threonine residue ^10^.

Thus, it is noted that certain functionally significant domains are much more conserved compared to the complete protein ^48^. In the current work, a methodology to annotate proteins in such ‘twilight’ zones ^36^ by fragmenting and aligning two protein sequences ( Figure 1) has been presented. The results are sorted based on differing criteria, and can be directed by a input file in case the sequences have already been annotated. This method helps in quickly honing onto conserved sites through visual inspection ( Table 1 and Figure 1). The threonine phosphorylation site (E=9.1e-4) for checkpoint kinases (Chk1/Chk2) ( Figure 1) and a new BRC repeat (E=17e-4) using FRAGAL ( Figure 2) has been identified. Pruning out matches which do not have a corresponding conserved sequence in hBRCA2 helps us to select fragment 61 in dmBRCA2 as a new BRC repeat ^7, 14^, and fragment 91 in dmBRCA2 as the putative threonine site for phosphorylation by checkpoint kinase Chk1 and Chk2 ^10^. It must be noted that the sites identified remain putative until verified by experimental data, in spite of the low E-values obtained.

The multiple alignments can be used to create (for the carboxy-terminal phosphorylation threonine site) or extend (for the new BRC repeat) Prosite motifs. However, the carboxy-terminal phosphorylation threonine site Prosite motif generated from the multiple alignment of sequences from *Drosophila* and mammals did not result in any matches in other organisms ( *Ustilago maydis* and *Caenorhabditis elegans*).

In order to justify this method further, I concentrated on proteins that contain the Half-a-tetratricopeptide (HAT) repeat motifs. The HAT motif is much less ubiquitous than the related tetratricopeptide (TPR) repeat, and has been implicated in various aspects of RNA metabolism ^23, 24^. HAT motifs are also hypothesized to play a critical role in assembling RNA-processing complexes ^25^. A recent study that combined bioinformatics, modeling and mutagenesis studies of the HAT domain used the three tandem HAT motifs in the *Saccharomyces cerevisiae* protein Utp6 to make inferences about the residues that confer structural and/or functional properties to the motif. In the current work, the detection of a new HAT repeat sequence (E=11e-4) in Utp6 from *S. cerevisiae* has been reported. This sequence has the desired aromatic residues at the proper spacing, a requisite for being considered a HAT repeat ^25^. The above mentioned study would have gained further by the knowledge of this HAT repeat, a repeat that remained undetected by sequence analysis using other methods. The HAT repeats are much more varied, and thus not suitable for generating motifs (like Prosite ^27^). For example, the consensus sequence has been derived from an alignment of 742 HAT motifs from Pfam ^30^ and had to be manually edited since this alignment included gaps in greater than 90% of the sequences ^25^. Moreover, FRAGAL detects that a particular HAT sequence in one protein is more related to HAT sequences from other species that other HAT repeats present in its own sequence. This raises interesting questions about their evolutionary history.

In some of the significant matches in Table 1 the fragment in hBRCA2 is not annotated to be functionally relevant - for example fragments 33 and 87 of dmBRCA2 and fragments 176 and 194 in hBRCA2, respectively. These fragments might suggest an important, yet unknown, functional relevance of that stretch of the human gene as well, since it is conserved across distant species. An excellent database for *Drosophila* related information is available at http://flybase.org/ ^54^. A database ( www.sanchak.com/fragal.html) for BRCA2 and nucleolar RNA-associated proteins from different organisms, and will be updating this on a regular basis to include more organisms and different repeats has been created. The increasing importance of *Drosophila* as a model system for cancer research ^55^ in the search for human therapeutics ^56– 58^ can be exploited to the hilt once the conserved mechanism is fully understood. FRAGAL presents the first step by annotating putative conserved sequence fragments in *Drosophila* and humans.
