# Supplementary material for: A fragmented alignment method detects a putative phosphorylation site and a putative BRC repeat in the Drosophila melanogaster BRCA2 protein
Source: F1000Res. 2013 Oct 7;2:143. Originally published 2013 Jun 25. [Version 2] doi: 10.12688/f1000research.2-143.v2 (PMC3924952; doi:10.12688/f1000research.2-143.v2)
Supplement: Peer review history

# Peer review: Referee response for version 2

**Published:** 2013-11-28

**Authors:** Saurabh Sinha

**Affiliations:** 1 Department of Computer Science, University of Illinois at Urbana-Champaign, IL, USA

I have read the author's rebuttal carefully, and am now completely satisfied with how he has addressed my previous comments.

I have read this submission. I believe that I have an appropriate level of expertise to confirm that it is of an acceptable scientific standard.

# Peer review: Referee response for version 2

**Published:** 2013-10-22

**Authors:** Satish Chikkagoudar

**Affiliations:** 1 Pacific Northwest National Laboratory, Washington, USA

I have read this submission. I believe that I have an appropriate level of expertise to confirm that it is of an acceptable scientific standard.

# Peer review: Referee response for version 1

**Published:** 2013-09-06

**Authors:** Saurabh Sinha

**Affiliations:** 1 Department of Computer Science, University of Illinois at Urbana-Champaign, IL, USA

In this well-written manuscript, Chakraborty presents a tool for local alignment of two protein sequences that includes a fragment-chaining step. He then uses this tool to identify important putatively functional fragments in two different *Drosophila *proteins by comparison to the respective human ortholog. A database containing results of many more similar applications is also presented and is a nice aspect of the work. 

I have the following specific comments, mostly related to the presentation, that might help the author improve the clarity of the manuscript.

The current title is rather long. The contribution of this work is mainly in the form of the FRAGAL tool, and the title could be trimmed to emphasize only that. The two example applications to finding the phosphorylation site, BRC repeat etc. are not experimentally substantiated biological claims, and may be better off being left out of the title. 

Clear discussion should be provided regarding other previous work where pairs of aligned fragments are stitched together (e.g. exon chaining, see Jones & Pevzner 2004, and the chain/net approach to whole genome alignments). 

Since the FRScore does not include gap penalties, I am assuming that each pair of fragments is subjected to two distinct similarity-scoring approaches; the gap-based approach when aligning that pair of fragments using 'needle' and the match/mismatch based approach when ranking the aligned pairs. This should be stated clearly, to avoid confusion. Is there a reason why the needle score was not used in place of the FRScore?

It appears that the FR score is the unweighted sum of %similarity and %identity. This should be stated explicitly.

I did not quite understand the formatting of Table 1.

- I think there should be a line separating ‘A’ from ‘B’ (which I think comes after a row with rank 14 in the second column). It took me some time to see that there are two sub-tables being shown here.
- Also, the fact that the same row is used to show different entities was confusing; usually a table is constructed so that a row shows different pieces of information about one entity.
- I assume something like A91-B337 refers to the starting positions of a matching fragment between sequences A and B, and the length of that fragment is not indicated in the row. Is this correct? (On reading further I realize that this interpretation is incorrect, and the numbers in a match are arbitrary indices and not coordinates. This was not clear from the legend.)
- I found that presenting sub-tables ‘A’ and ‘B’ (which I finally realized does not relate to ‘A’ and ‘B’ sequences) leads to more confusion than it helps. If both sub-tables present the same ranked list and the only difference is that ‘A’ filters for “conserved” fragments, then it might be better to show only sub-table ‘B’ and add a column indicating if this is a fragment marked conserved.

Where is the E-value of an FRScore coming from? (I am not familiar with what the ' *Protein Information Resource*' provides.) Perhaps this E-value correspond to the global alignment score reported by needle?

The results of Table 1 do not aid ones understanding as to how the fragmented alignment, i.e. stitching together of fragments, helps in this case. As shown, this appears similar in form to a ranked list of matches from a standard local-aligner. Similarly, with respect to Figure 2, the author may wish to discuss why FRAGAL finds the 'melanogaster4' fragment as a BRC repeat where previous annotations (that found three repeats) failed. Was this a matter of previous methods ‘ *missing the threshold*’? (This seems unlikely given the strong E-value reported for this.) Similar clarifications for the HAT repeat finding exercise will also be helpful.

I have read this submission. I believe that I have an appropriate level of expertise to confirm that it is of an acceptable scientific standard.

# Response

**Published:** 2013-09-17

**Authors:** Sandeep Chakraborty

**Affiliations:** Tata Institute of Fundamental Research, India

I greatly appreciate the positive comments.

*The current title is rather long. The contribution of this work is mainly in the form of the FRAGAL tool, and the title could be trimmed to emphasize only that. The two example applications to finding the phosphorylation site, BRC repeat etc. Are not experimentally substantiated biological claims, and may be better off being left out of the title.*

 

Since this work began in search for unannotated fragments of the dmBRCA2 sequence, and is being followed in the lab actively, I think the reference to an application of FRAGAL is warranted. However, I have removed the reference to the HAT repeat and stated the fact that the phosphorylation site and the BRC repeat is putative in the title.

*Clear discussion should be provided regarding other previous work where pairs of aligned fragments are stitched together...*

I have discussed the spliced alignment approach to genome assembly in the discussion. However, I have noted that while these methods use graph algorithms to solve the computationally difficult problem of exon chaining, FRAGAL does the converse by finding best matches in known exon chains (i.e. protein sequences).

*Since the FRScore does not include gap penalties, I am assuming that each pair of fragments is subjected to two distinct similarity-scoring approaches; the gap-based approach when aligning that pair of fragments using 'needle' and the match/mismatch based approach when ranking the aligned pairs. This should be stated clearly, to avoid confusion. Is there a reason why the needle score was not used in place of the FRScore?*

 

The needle score includes gap penalties, which is something that is not intended for use in FRScore, as you have correctly pointed out. The idea is to direct the alignment to discourage gaps – but once the alignment is done a gap should not have a penalty. It is ‘real' and therefore only the identity or similarity that matters.

*It appears that the FR score is the unweighted sum of %similarity and %identity. This should be stated explicitly*.

I have empirically assigned more weightage to the %identity based on the fact that we are searching for repeats, and expect higher conservation. This conservation is magnified a bit more by assigning higher weightage.

*I did not quite understand the formatting of Table 1. ... I found that presenting sub-tables A and B (which I finally realized does not relate to A and B sequences) leads to more confusion than it helps.*

I apologize for this confusion. The line demarcating subtables A and B was lost in the typesetting - and I missed out on detecting this error. Further, naming the subtables A and B was a poor choice of names, since the sequences were also named A and B. Finally, I agree that the second column was unnecessary, as was two subtables. I have simplified this table.

*I assume something like A91-B337 refers to the starting positions of a matching fragment between sequences A and B, and the length of that fragment is not indicated in the row. Is this correct? (On reading further I realize that this interpretation is incorrect, and thenumbers in a match are arbitrary indices and not coordinates. This was not clear from the legend.)*

I apologize for this oversight. This is mentioned in the web pages - http://sanchak.com/fragal/ALLRUNS.BRCA2/Caenorhabditiselegans.G5EG86.Homosapiens.P51587.gap.25.size30/cumu.results.thresh30.size30.doiden1.onlyanno1.html ****as ` *Multiply index with 10 to get sequence* ****** *starting position in original sequence* *'*. Thus A91 refers to the sequence starting at 910 in `A' and ****going till 959, since the fragmenting length is 50. I have now mentioned this at the beginning of ****the Results section, and in the legend.

*Where is the E-value of an FRScore coming from? ... Perhaps this E-value corresponds to the global alignment score reported by needle?*

I have specified the website ( http://pir.georgetown.edu/pirwww/search/pairwise.shtm ), and cited the paper by Wu C *et al.* (2003) The Protein Information Resource. Nucleic Acids Res 31: 345347.

*... with respect to Figure 2, the author may wish to discuss why FRAGAL finds the *'melanogaster4' *fragment as a BRC repeat where previous annotations (that found three repeats) failed. ... Similar clarifications for the HAT repeat finding exercise will also be helpful.*

I could only make an educated guess as to why other tools failed to detect these repeats. I believe that the tools used had a ‘sequential' methodology and therefore one match fixed the order of the next searches. Not all known BRC repeats have a low E-value when aligned with the new BRC repeat. For example, the first BRC repeat in hBRCA2 when aligned to the new dmBRCA2 repeat has an E=0.04, much more than the E=17e-4 observed for the fourth repeat (which is the one I report here). Ideally, if one took all the BRC repeats and did a search in the dmBRCA2 sequence, this new repeat would be reported. Essentially, this is what FRAGAL does, albeit implicitly, by automatically fragmenting the sequence. The same logic applies to the HAT repeat, where the sequences are more varied and thus the choice of the repeat would affect the detection of new motifs.

# Peer review: Referee response for version 1

**Published:** 2013-08-22

**Authors:** Satish Chikkagoudar

**Affiliations:** 1 Pacific Northwest National Laboratory, Washington, USA

The author presents an interesting technique for detecting new BRC repeats. The paper is generally well written, but needs some additional material to bolster its case. The introduction section needs more discussion of the 'state-of-the-art' in the alignment and motif detection area (especially with respect to detecting BRC repeats). The paper’s argument can be made stronger by explicitly mentioning the advantages of fragmented alignment over any other recursively applied local alignment  method or homology search method. Some discussion of existing methods exists in the discussion/results section, but that needs to be made available in the introduction in order to justify the need for creating a new method An explanation of the choice of parameter values needs to be given. For example, why does FRscore have weights of 1/3 for only Similarity (equation 2)? Also, the reasoning for choosing particular values for gap open and gap extend penalties needs to be mentioned along with whether parameter tuning/search was done to arrive at those values. Otherwise, those numbers seem arbitrary. The author needs to discuss whether he tried any other alignment algorithms apart from Clustal-W. Some other algorithms such as MAFFT, ProbCons or CONTRAlign may yield better results. The author may want to discuss the data in a separate section under methods/materials. A figure describing the FRAGAL pipeline will be useful to visually describe the pipeline/algorithm.   

I have read this submission. I believe that I have an appropriate level of expertise to confirm that it is of an acceptable scientific standard, however I have significant reservations, as outlined above.

# Response

**Published:** 2013-08-23

**Authors:** Sandeep Chakraborty

**Affiliations:** Tata Institute of Fundamental Research, India

Dear Dr Chikkagoudar,

I would like to thank you for your insightful suggestions which will help improve the manuscript. I will make the suggested changes, and incorporate them in a new version shortly.

A small clarification - you have asked for a "figure describing the FRAGAL pipeline". There is a supplementary figure S1 doing this. Do you think that this figure is insufficient, or were you suggesting that I move this to the main manuscript?

Best regards,

Sandeep

# Response

**Published:** 2013-09-17

**Authors:** Sandeep Chakraborty

**Affiliations:** Tata Institute of Fundamental Research, India

I appreciate the positive comments, and hope to have addressed the concerns detailed below. 

*The introduction section needs more discussion of the 'state-of-the-art' in the alignment and motif detection area (especially with respect to detecting BRC repeats) ... Some discussion of existing methods exists in the discussion/results section, but that needs to be made available in the introduction in order to justify the need for creating a new method.*

 I have moved this section to the introduction. In response to the comments of another reviewer (Dr Saurabh Sinha), I have also mentioned the possible reasons why existing tools have failed to detect these repeats. Further, I have cited methods that use spliced alignment methods for genome assembly, noting that these methods use graph algorithms to solve the computationally difficult problem of exon chaining. FRAGAL does the converse by finding best matches in known exon chains (i.e. protein sequences). 

*An explanation of the choice of parameter values needs to be given. For example, why does FRscore have weights of 1/3 for only Similarity (equation 2)?*

 The extra weightage given to % identity in the score is due to the fact that one expects more sequence conservation in repeats. 

*...the reasoning for choosing particular values for gap open and gap extend penalties needs to be mentioned along with whether parameter tuning/search was done to arrive at those values.*

 The gap penalties are set to discourage gaps, but not gap extensions. The gap opening has been set to two values - 10 and 25. Results using both values have been uploaded in the database http://sanchak.com/fragal/BRCA2.html . The results using 25 have been observed to be better. However, a complete statistical analysis of these values is beyond the scope of this work. 

*The author needs to discuss whether he tried any other alignment algorithms apart from Clustal-W. Some other algorithms such as MAFFT, ProbCons or CONTRAlign may yield better results.*

 I have used another tool (MAFFT) to generate the multiple sequence alignment. The results from the new alignment tool, mirrors the inference drawn from Clustal-W. This is now a supplementary figure. 

*A figure describing the FRAGAL pipeline will be useful to visually describe the pipeline/algorithm*. 

 I have added a pseudo code of the FRAGAL program in the main manuscript.

# Peer review: Referee response for version 1

**Published:** 2013-08-12

**Authors:** Himanshu Sinha

**Affiliations:** 1 Department of Biological Sciences, Tata Institute of Fundamental Research, Mumbai, India

A well written paper that proposes a new method, FRAGAL for identifying functional putative motifs within protein sequences which have been hidden from previous analyses. By splitting the sequences into overlapping fragments, this method is able to discover additional motifs. The author has tested his technique on BRC repeats in *Drosophila dm*BRCA2 **and HAT repeats in budding yeast Utp6, by comparing them to corresponding human protein sequences. The BRC repeat has been well analysed with comparisons across several *Drosophila* species. However the author does not provide extensive comparison of HAT repeats in *Saccharomyces* species. Since the sequences of several *Saccharomyces *sibling species and closely related fungi such as *Aspergillus*, *Candida*, etc. are known, it would be interesting to see how conserved this new HAT repeat is within the overall conservation of Utp6. 

While the author establishes the advantage of FRAGAL technique, it is too early to say that this is a useful generic tool to identify known and novel motifs in protein sequences. I would request the author to run his FRAGAL code on several protein sequences with small motifs to estimate success rates and false discovery rates of his method. A supplementary table should be provided describing several sequences analysed by this method and these rates. 

A minor comment, Table 1 should be simplified with the two BRCA2 protein sequences presented in two sub-tables. Please explain why certain ranks are missing in FRscore and %S.

I have read this submission. I believe that I have an appropriate level of expertise to confirm that it is of an acceptable scientific standard.

# Response

**Published:** 2013-08-23

**Authors:** Sandeep Chakraborty

**Affiliations:** Tata Institute of Fundamental Research, India

Dear Dr Sinha,

I greatly appreciate your comments on my manuscript. I will incorporate your suggested changes and update the manuscript. Your suggestion of including more of such repeats (I plan to do BIR and TPR) is something that will take some computational time and thus the delay.

Best regards,

Sandeep

# Response

**Published:** 2013-09-17

**Authors:** Sandeep Chakraborty

**Affiliations:** Tata Institute of Fundamental Research, India

I am grateful for the encouraging comments on the work. 

*...author does not provide extensive comparison of HAT repeats in *Saccharomyces *species. ... it would be interesting to see how conserved this new HAT repeat is within the overall conservation of Utp6.*

I have implemented this interesting idea using proteins which have the HAT repeat from *Aspergillus nidulans* and *Candida glabrata*. This is now a Supplementary figure. However, these do not provide any further insights into the evolution of the HAT repeat, and would require sophisticated analyses beyond my expertise. 

*I would request the author to run his FRAGAL code on several protein sequences with small motifs to estimate success rates and false discovery rates of his method. A supplementary table should be provided describing several sequences analyzed by this method and these rates.*

In accordance with this suggestion, I have run FRAGAL on two more motifs (BIR and TPR). However, I failed to detect any new repeats using these two motifs. These are now part of the database - http://sanchak.com/fragal.html. 

*A minor comment, Table 1 should be simplified with the two BRCA2 protein sequences presented in two sub-tables. Please explain why certain ranks are missing in FRscore...*

I have simplified the table considerably based on the comments of another reviewer (please see below). The naming of the sub tables as A and B was confusing given that the query and target sequences were named A and B. Further; the columns for the similarity scoring has been removed. We did not ever use the similarity only score, and this was adding to the confusion. I have now clearly stated the reason for some missing ranks. I apologize for the confusing aspects of this table.
